# Broncholithiasis mimicking metallic projectiles at autopsy: a forensic diagnostic pitfall in putrefied bodies

**DOI:** 10.1007/s00414-026-03916-9

**Published:** 2026-07-07

**Authors:** Aymeric Zambiasi, Eulalie Pefferkorn, Fabrice Dedouit, Claudie Josse, Norbert Telmon, Lise Pestourie

**Affiliations:** 1 Institute of Legal medicine, Purpan Hospital, Place du Docteur Joseph Baylac, Toulouse, 31059 France; 2 Université de Toulouse, 118 route de Narbonne, Toulouse Cedex, 31062 France; 3 Centre de Microcaractérisation Raimond Castaing, Clément Ader Institute, 3 Rue Caroline Aigle, Toulouse, 31400 France

**Keywords:** Broncholithiasis, Post-mortem computed tomography, SEM-EDS, Putrefaction

## Abstract

We report an autopsy case in which broncholithiasis mimicked retained ballistic projectiles in a putrefied body. A medico-legal autopsy was performed on a 76-year-old male found in an advanced state of putrefaction. Macroscopic examination during autopsy revealed small, stony intrapulmonary nodules raising suspicion of retained pellets. Whole-body post-mortem computed tomography (PMCT) allowed us to further characterize these elements. The structures were subsequently analyzed using scanning electron microscopy coupled with energy-dispersive X-ray spectroscopy (SEM-EDS) to determine their elemental composition. PMCT demonstrated multiple hyperdense micronodules within the right upper lobe, arranged in a linear endobronchial pattern without metallic artifacts, strongly suggesting a calcified biological origin. SEM-EDS analysis revealed a predominance of calcium and phosphorus, with no evidence of metallic elements typically associated with ballistic materials. These findings and histopathological examination confirmed the diagnosis of broncholithiasis. Broncholithiasis may closely mimic retained ballistic fragments at the macroscopic level in decomposed bodies, representing a significant forensic diagnostic pitfall. While PMCT is key to orienting the diagnosis, this work highlights the value of a multimodal approach combining post-mortem imaging, histopathology, and elemental microanalysis to provide definitive chemical confirmation and prevent misinterpretation of the cause and manner of death.

## Introduction

The medicolegal examination of bodies in an advanced state of decomposition or mummification presents significant challenges. The degradation of soft tissues and the loss of anatomical landmarks can obscure wound tracks and mimic traumatic injuries, often leading to diagnostic bias. In such contexts, post-mortem computed tomography (PMCT) can be particularly useful to diagnose lesions that can be macroscopically overlooked and can help to guide the autopsy of putrefied bodies [1, 2]. In such cases, the identification of dense, radio-opaque structures within body cavities requires careful evaluation to distinguish between endogenous biological processes and exogenous foreign bodies, such as ballistic projectiles. Misinterpretation may lead to major errors in determining the cause and manner of death.

Broncholithiasis is a rare condition characterized by the presence of calcified or ossified material (broncholiths) within the bronchial lumen [3–6]. Although typically associated with chronic granulomatous diseases, it is rarely encountered in forensic practice and may be unexpected at autopsy. Its macroscopic appearance may closely mimic metallic foreign bodies, particularly in altered post-mortem conditions, thereby representing a potential source of diagnostic error.

We report a case in which broncholithiasis mimicked retained ballistic pellets in a putrefied body. Definitive identification was achieved through a multimodal approach combining PMCT and elemental microanalysis via scanning electron microscopy coupled with energy-dispersive X-ray spectroscopy (SEM-EDS), highlighting a critical forensic diagnostic pitfall and emphasizing the value of complementary analytical techniques in complex medico-legal investigations.

## Case report

### Case history and autopsy findings

A 76-year-old male was found deceased at his residence in a state of advanced putrefaction. No reliable medical history was available. A medico-legal autopsy was ordered to establish the cause and manner of death.

External examination revealed marked putrefactive changes, including diffuse brownish and greenish skin discoloration associated with near-total mummification. Numerous pupae, along with a few maggots and dead flies, were present on the body. No wounds, recent traumatic lesions, or distinctive integumentary findings were identified.

**Fig. 1:**
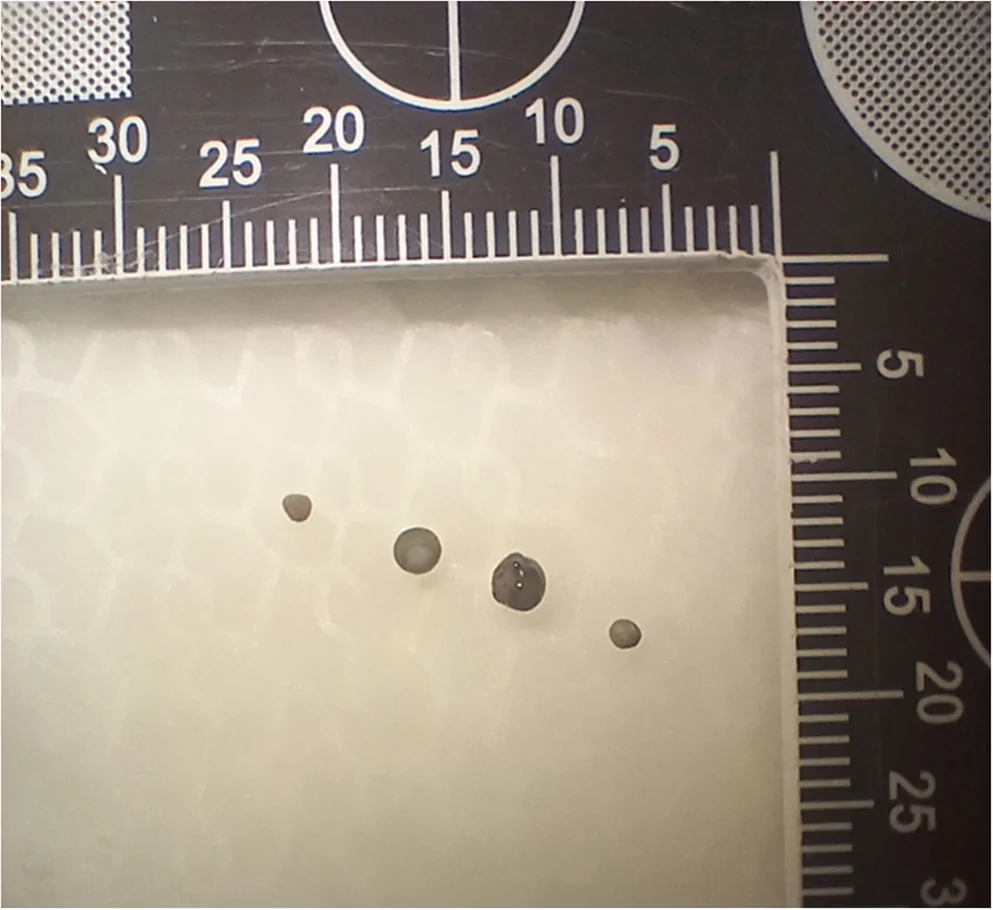
Macroscopic appearance of the four round-shaped grey colored, recovered from the upper lobe of the right lung during autopsy resembling ballistic pellets

All internal organs exhibited severe putrefactive changes. Metallic rib cerclage wires were noted on the left thoracic wall, alongside pleuropulmonary adhesions, indicating prior thoracic surgery or trauma. Diffuse aortic and coronary calcification were observed. The anterior interventricular artery and the abdominal aorta showed atherosclerotic plaques without significant stenosis or obstruction of the lumen. Macroscopic dissection of the right lung discovered four greyish elements with a stony consistency in the upper lobe, irregular, and spherical, ranging from 1 to 2 mm of diameter (Fig. 1). Due to advanced putrefactive changes, their precise anatomical location and distribution within the pulmonary parenchyma could not be reliably assessed.

Given the unexplained nature of the death and the advanced putrefaction limiting the autopsy findings, the possibility of retained projectiles was considered. This hypothesis was further supported by the evidence of prior thoracic trauma, despite the absence of acute internal hemorrhage. In addition, the lack of recent cutaneous disruption was consistent with the presence of past retained ballistic pellets rather than a recent gunshot wound.

### PMCT findings

**Fig. 2:**
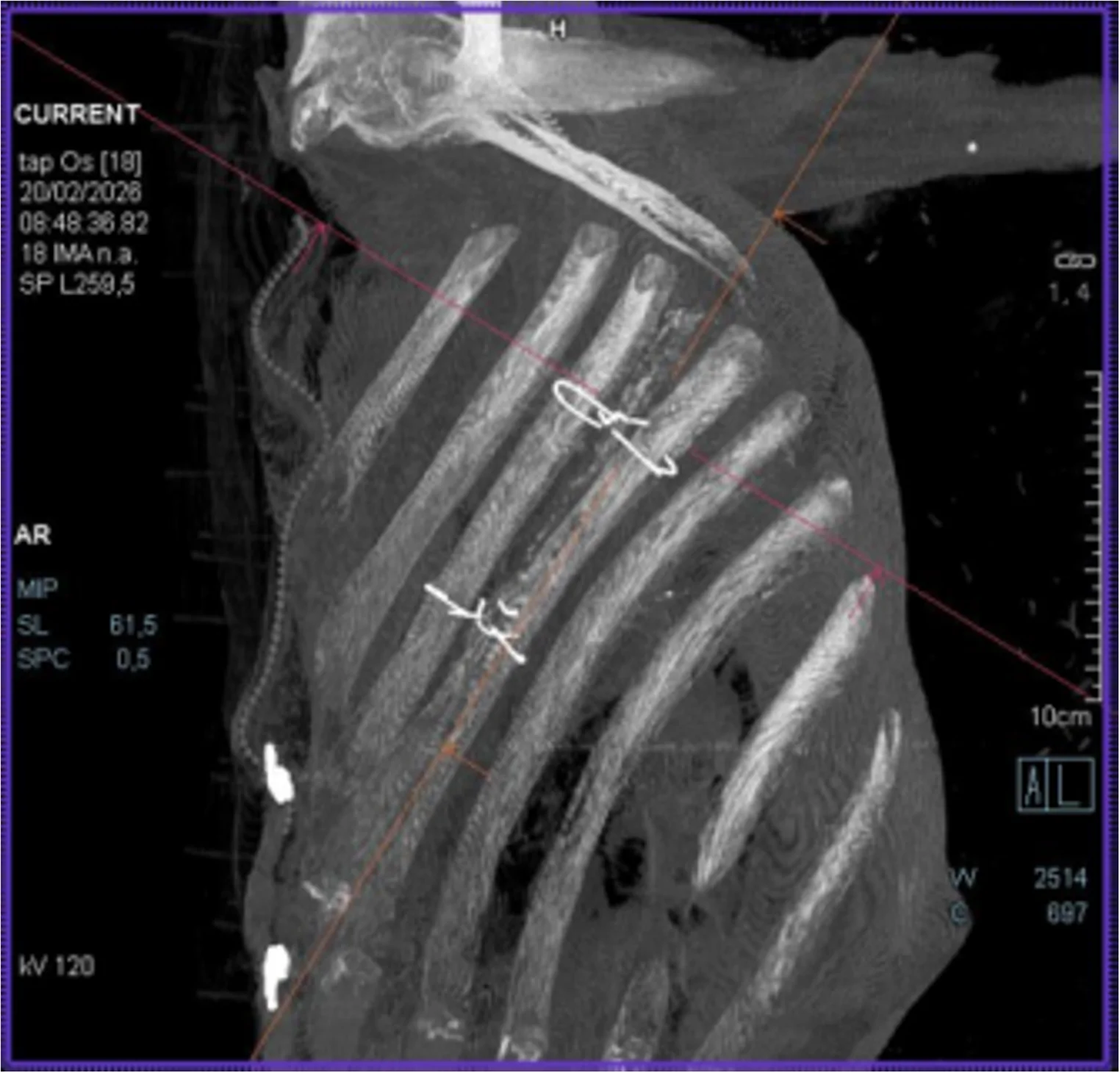
PMCT MIP (Maximum Intensity Projection) reconstruction, sagittal view. Visualization of the metallic wires around the 5th and 6th lateral arcs of the left ribs

**Fig. 3:**
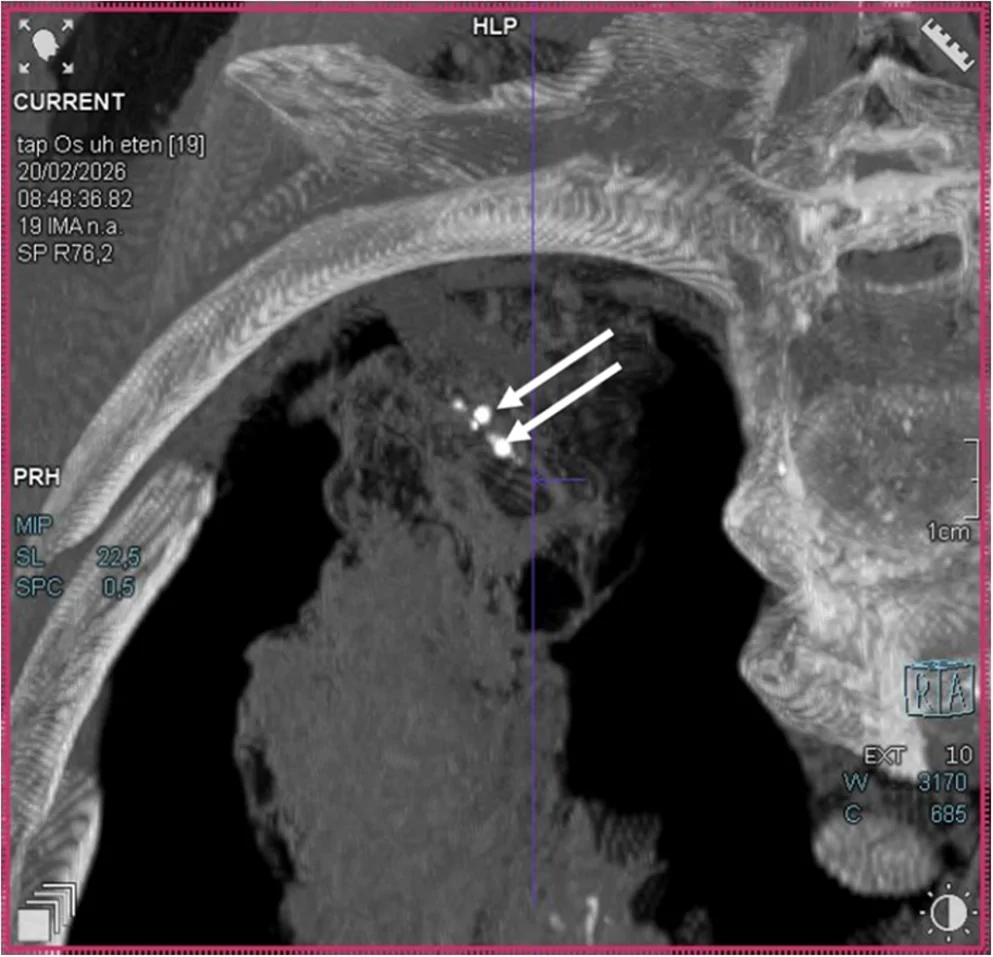
PMCT MIP (Maximum Intensity Projection) reconstruction, coronal view. Visualization in the putrefied pulmonary parenchyma of 4 round-shaped hyperdense elements (arrows). The mean density was around 3000 Hounsfield Units for each of them

Full-body PMCT was performed (Siemens Definition AS 20, Erlangen, Germany) with bone and soft tissues kernels, slice thickness: 0.75 mm, 120 kV, 89 mAs. Pre-autopsic analysis of the PMCT established the generalized atherosclerosis and the presence of surgical cerclage wires as observed during the autopsy (Fig. 2). No radiologic evidence of recent traumatic lesion was identified. After the autopsy, a retrospective review of the PMCT data, with focused multiplanar reconstructions of the right upper pulmonary lobe demonstrated four intraparenchymal, hyperdense micronodular elements, arranged in a linear pattern consistent with an endobronchial distribution (Fig. 3). No beam-hardening or metallic streak artifacts were observed surrounding these elements. The mean density was around 3000 Hounsfield Units, confirming the calcified nature of the elements. These radiological findings suggested a biological, calcified origin—likely secondary to a granulomatous process—rather than exogenous metallic material.

### SEM-EDS analysis

To definitively characterize the chemical composition of the recovered structures, the samples were analyzed using SEM-EDS. This analysis was conducted at the Raimond Castaing Microcharacterization Center in Toulouse. A Thermofisher Helios Nanolab 600i microscope equipped with an electron backscatter diffraction (EBSD) detector and an energy-dispersive X-ray (EDS) detector was used. The analysis was performed in high-vacuum mode. To avoid any interference with the support, the samples were placed on carbon stubs covered with carbon adhesive tape.

**Fig. 4:**
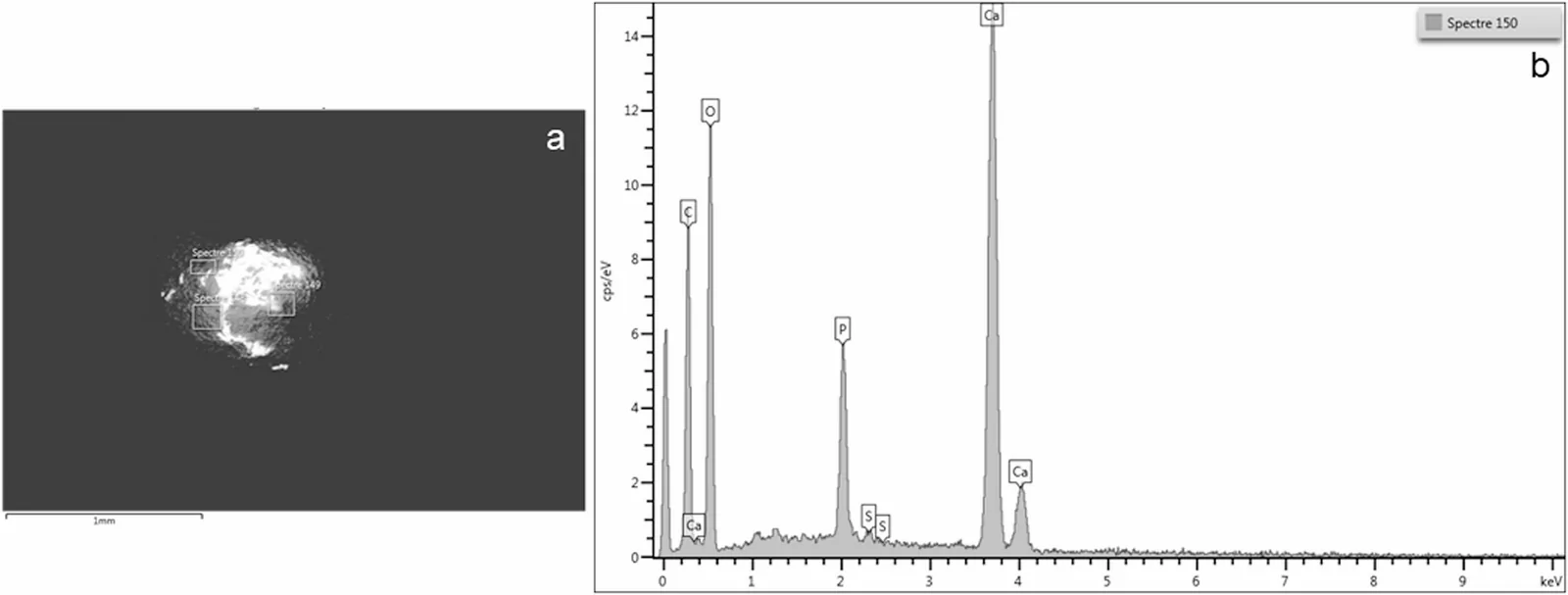
SEM-EDS analysis of the pulmonary concretions a: Scanning Electron Micrograph of one of the four irregular stony elements recovered at autopsy b: EDS spectrum showing the mineral composition of the concretion (Ca, P) evocative of a biological process

This analysis demonstrated a predominant calcium (Ca) peak associated with a lower phosphorus (P) peak (Fig. 4). Notably, no metallic elements typically associated with ballistic alloys, such as lead (Pb), antimony (Sb), barium (Ba), copper (Cu) or iron (Fe), were detected. Furthermore, no magnesium or silica was identified. This elemental profile was consistent with calcium phosphate-based mineralization, supporting a biological origin.

### Histopathological examination

**Fig. 5:**
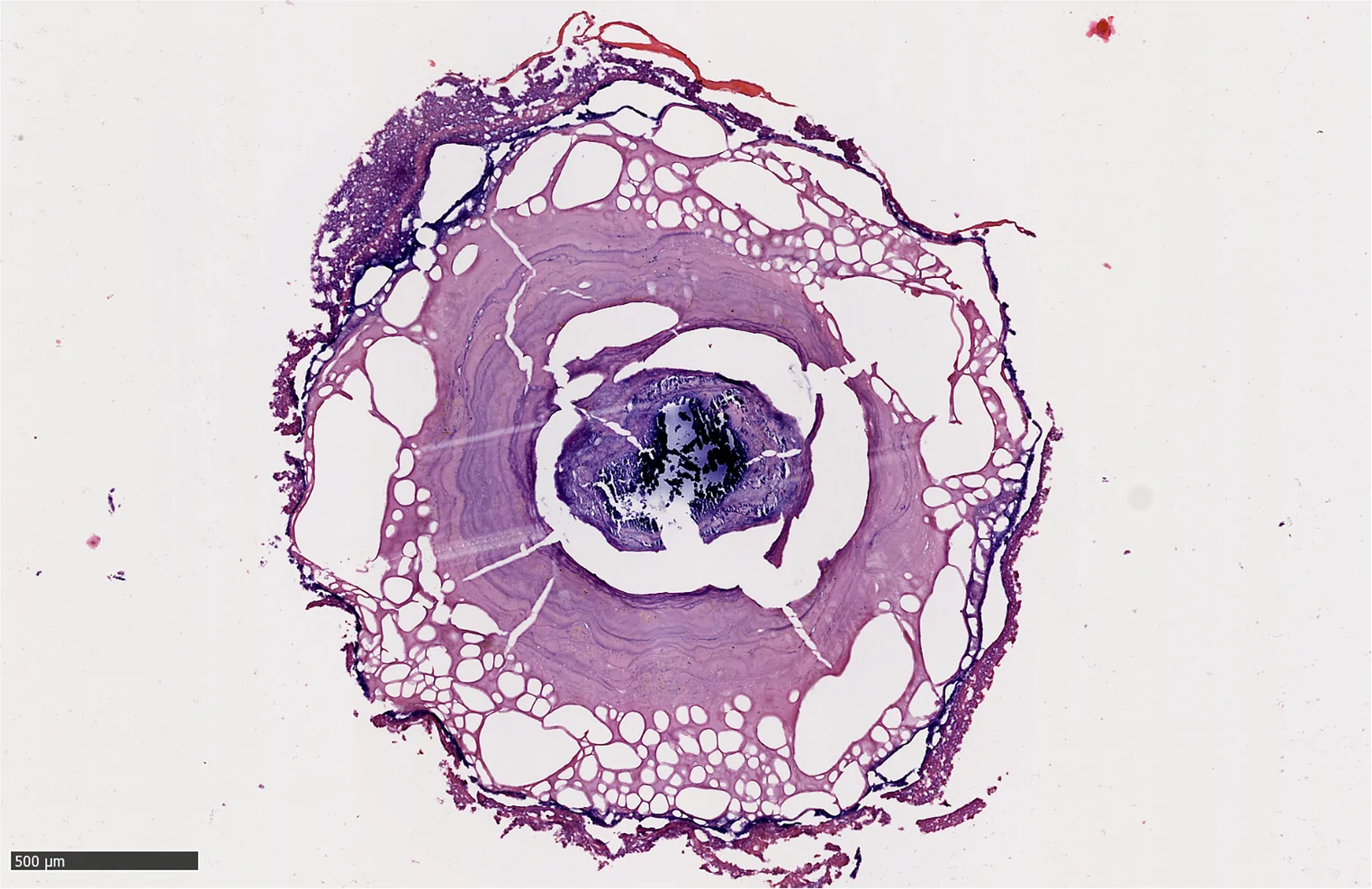
Histological paraffin-embedded cross section (hematoxylin and eosin staining) of one of the recovered structures showing a calcified core surrounded by mineralized concentric lamellar deposits and decalcification artifacts in the outer part of the sample

Following a brief decalcification step, microscopic examination of one the recovered structures was performed to characterize their architecture. The sections revealed a calcified core surrounded by mineralized concentric lamellar deposits. No residual cellular structures or microorganisms were identified. Decalcification artifacts were present in the outer part of the sample (Fig. 5). These morphological features were indicative of dystrophic calcification of biological origin.

### Case conclusion

The integration of these modalities allowed for a clear diagnostic progression. While the retrospective PMCT density and topographical analysis strongly oriented the diagnosis toward a benign biological calcification, the SEM-EDS elemental microanalysis and subsequent histopathological examination provided definitive confirmation of broncholithiasis. Ultimately, no evidence of ballistic injury or recent trauma was identified. These broncholiths were considered incidental findings and did not play a role in the lethal mechanism. Following the medico-legal autopsy and the application of adjunct analytical techniques, the cause of death remained officially undetermined, although the findings were compatible with a natural death.

## Discussion

This case illustrates a significant diagnostic pitfall in forensic pathology: the potential misinterpretation of broncholithiasis as retained ballistic fragments within a putrefied body.

Broncholithiasis has an estimated incidence of 0.1% to 0.2% among all lung diseases [6]. It is thought to arise from the erosion of a calcified peribronchial lymph node through the bronchial wall into the adjacent lumen, most frequently as a late sequela of granulomatous infections such as tuberculosis or histoplasmosis [3–7]. The most common locations are the middle and right upper and pulmonary lobes, as observed in our case [4–6]. While often asymptomatic, broncholithiasis may cause cough, lithoptysis, recurrent infections, hemoptysis, and the formation of fistulae with mediastinal structures [3–7].

While broncholithiasis is occasionally documented in clinical autopsies where pre-mortem imaging is available [8, 9], its description in a forensic setting remains exceptional, despite the significant diagnostic challenges it poses [10]. In a medicolegal context, the identification of dense intrathoracic structures during autopsy or imaging may raise suspicion of foreign bodies, particularly in cases of suspected violent death. Metallic projectiles from firearms are among the primary considerations in such contexts. This hypothesis may be further strengthened by contextual elements such as prior trauma or surgical history.

Furthermore, decomposition-related changes can significantly alter tissue consistency and obscure anatomical relationships, making traditional gross examination unreliable. In such cases, adjunctive techniques are essential to ensure accurate diagnosis and avoid misinterpretation.

Several factors contributed to the initial misinterpretation in this case: the advanced decomposition of the body and organs, the macroscopic stony and metallic-like appearance of the broncholiths, and the presence of rib cerclage wires with pleuropulmonary adherence, suggesting prior trauma and surgery, thereby raising suspicion of possible firearm involvement. Together, these elements created a misleading forensic context in which broncholithiasis mimicked ballistic remnants. Such elements can bias forensic interpretation, especially when findings are assessed in isolation as in our case where no clinical history was available in this context of unexplained death which made the interpretation of the per-autopsy findings even more challenging.

Post-mortem computed tomography is now widely used in forensic practice to detect and characterize foreign bodies and assess trauma [11, 12]. In our case, the retrospective review of the PMCT data played a decisive role in resolving the macroscopic ambiguity encountered during the autopsy. The spatial distribution was consistent with an endobronchial topography of the stony elements, while the measured density (around 3000 HU) and the complete absence of beam-hardening or metallic streak artifacts strongly argued against projectile fragments. This highlights that the primary diagnostic pitfall occurred at the macroscopic level during gross dissection, where putrefactive changes distorted the tissues, rather than at the radiological level, where the PMCT findings were highly suggestive of a calcified biological process.

Advanced analytical techniques such as SEM-EDS are increasingly used in forensic science for material identification and can be useful in forensic investigations as it enables precise characterization of chemical composition [13–15]. While the PMCT findings provided strong diagnostic evidence favoring a biological origin, SEM-EDS analysis provided valuable, objective chemical confirmation. Broncholiths are typically composed of calcium salts, predominantly calcium phosphate [6–8] consistent with dystrophic calcification processes [16]. Less commonly, they can be composed of magnesium oxide [17]. The SEM-EDS findings in this case (dominant calcium with associated phosphorus) are consistent with previous descriptions of mineral composition of broncholiths. The use of SEM-EDS provided objective chemical confirmation of the biological, non-metallic nature of the material, which was critical for the formal exclusion of ballistic fragments.

The differential diagnosis for such hyperdense intrapulmonary structures in forensic cases includes granulomatous infections (such as tuberculosis), pneumoconiosis, calcified granulomas, or metastatic calcifications secondary to metabolic disorders. In the present case, pneumoconioses like silicosis or anthracosilicosis were ruled out due to the absence of silica, or other mineral dusts on the EDS spectrum [18]. Furthermore, the localized, non-diffuse nature of the four nodules, restricted to a linear endobronchial distribution in the right upper lobe, was structurally inconsistent with systemic metabolic calcifications or diffuse occupational lung diseases [19]. This specific anatomical restriction, combined with the concentric lamellar architecture observed on histopathology, formally pointed to broncholithiasis secondary to an old, localized granulomatous process. Failure to correctly identify broncholithiasis could have resulted in incorrect conclusions regarding the cause and manner of death, potentially triggering unnecessary criminal investigation. In general, awareness of broncholithiasis as a medical entity and its proper identification is essential for forensic pathologists and radiologists as it can accompany several diseases or explain certain lung features at autopsy.

This case underscores the importance of a systematic, multimodal approach combining autopsy findings, imaging, and analytical techniques to avoid misinterpretation, particularly in decomposed bodies where conventional examination is limited.

## Conclusion

Broncholithiasis is a rare but important incidental finding at autopsy. Its macroscopic appearance can closely mimic metallic foreign bodies, therefore representing a substantial forensic diagnostic pitfall, especially in decomposed bodies. This case highlights the crucial role of PMCT in the assessment of autopsy findings, particularly when putrefactive changes limit traditional gross examination. Furthermore, it underscores the added value of elemental microanalysis via SEM-EDS in providing definitive confirmation of material composition. Awareness of this entity and the use of complementary analytical methods are essential to prevent misinterpretation and ensure accurate medico-legal conclusions regarding the cause and manner of death.

## Data Availability

No datasets were generated or analysed during the current study.
